# A time slice analysis of dentistry students’ visual search strategies and pupil dilation during diagnosing radiographs

**DOI:** 10.1371/journal.pone.0283376

**Published:** 2023-06-08

**Authors:** Conrad Borchers, Thérése F. Eder, Juliane Richter, Constanze Keutel, Fabian Huettig, Katharina Scheiter

**Affiliations:** 1 Eberhard Karls University of Tübingen, Tübingen, Germany; 2 Leibniz-Institut für Wissensmedien, Tübingen, Germany; 3 Department of Oral- and Maxillofacial Radiology, Centre for Dentistry, Oral Medicine, and Maxillofacial Surgery at the University Hospital Tübingen, University of Tübingen, Tübingen, Germany; 4 Department of Prosthodontics, Centre for Dentistry, Oral Medicine, and Maxillofacial Surgery at the University Hospital Tübingen, University of Tübingen, Tübingen, Germany; University of the Pacific Arthur A Dugoni School of Dentistry, UNITED STATES

## Abstract

Diagnosing orthopantomograms (OPTs: panoramic radiographs) is an essential skill dentistry students acquire during university training. While prior research described experts’ visual search behavior in radiology as global-to-focal for chest radiographs and mammography, generalizability to a hybrid search task in OPTs (i.e., searching for multiple, diverse anomalies) remains unclear. Addressing this gap, this study investigated visual search of N = 107 dentistry students while they were diagnosing anomalies in OPTs. Following a global-to-focal expert model, we hypothesized that students would use many, short fixations representing global search in earlier stages, and few, long fixations representing focal search in later stages. Furthermore, pupil dilation and mean fixation duration served as cognitive load measures. We hypothesized that later stages would be characterized by elaboration and a reflective search strategy, leading to higher cognitive load being associated with higher diagnostic performance in late compared to earlier stages. In line with the first hypothesis, students’ visual search comprised of a three-stage process that grew increasingly focal in terms of the number of fixations and anomalies fixated. Contrary to the second hypothesis, mean fixation duration during anomaly fixations was positively associated with diagnostic performance across all stages. As OPTs greatly varied in how difficult it was to identify the anomalies contained therein, OPTs with above-average difficulty were sampled for exploratory analysis. Pupil dilation predicted diagnostic performance for difficult OPTs, possibly capturing elaborative cognitive processes and cognitive load compared to mean fixation duration. A visual analysis of fine-grained time slices indicated large cognitive load differences towards the end of trials, showcasing a richness-resolution-trade-off in data sampling crucial for future studies using time-slicing of eye tracking data.

## Introduction

Reading orthopantomograms (OPTs, panoramic radiographs of the upper and lower jaw including dentition) is an integral part of dentistry training in higher education. In dentistry, OPTs are frequently employed to reveal a wide range of maladies and inform further diagnostic procedures and treatment. To date, only few research exists about the visual search processes in OPTs that are necessary to detect anomalies. Research in medical imaging areas (e.g., mammography) described visual search as a global-to-focal process [1]. However, it is unclear if insights from mammography or chest radiographs, which typically contain only a few anomalies [2, 3], apply to visual search of OPTs, which typically contain many more anomalies, pertaining to the gums, jaw, or teeth of patients [4]. Some studies provide evidence that visual search in OPTs also follows a global-focal-search [5, 6], while others do not [7]. Given this mixed evidence, the present study uses eye tracking to investigate with time slice analysis whether dentistry students’ interpretation of OPTs follows a global-to-focal search. The contribution of this study is three-fold: First, with the chosen methodology we provide a better understanding of visual search in OPTs as it develops over time. Second, our sample gives insight into the visual search strategies of dentistry students, thereby complementing research on medical image processing in experts. Third, we offer further evidence regarding how cognitive load changes during visual search of OPTs and how it is associated with the detection of anomalies. In the following, we first provide insights into the use of eye tracking technology to study visual search processes before addressing the process of visual search and cognitive load.

### Eye tracking for radiograph investigation

Eye tracking opens new possibilities for studying the visual search strategies of dentistry students. Eye tracking allows measuring gaze locations and movement on a 2-D plane at a fine-grained spatial and temporal resolution. Eye tracking measures can be classified into position measures (Where did the participant look?), movement measures (Where and how did the eye move?), and numerosity measures (At what rate did gaze measures occur?) [8]. Frequently used f features of gaze behavior include the number of fixations (i.e., the number of times the gaze is resting for a time period exceeding a certain threshold. e.g., 50 ms), the average duration of fixations, and saccades (rapid transitions between fixations as indicated by eye movements occurring at a speed of more than 75 visual degrees per second). In this study, we employ the number and average duration of fixations to characterize the learner’s visual search during the diagnosis of radiographs. Importantly, these measures are frequently defined and aggregated for *areas of interest* (AOIs), which are specific regions or objects relevant to the investigated subject matter. AOIs can represent relevant cues to technical failures in aviation training [9] and the disclosure of sponsored content in marketing [10] and anomalies in radiographs (for review see: [11]). In this study, we analyzed eye tracking measures (e.g., number of fixations) obtained for multiple areas of interest (AOIs) in each radiograph. These AOIs represent anomalies (e.g., symptoms indicating a calcification of the carotid artery, bone loss or caries) that were identified by two experts who diagnosed each radiograph. This definition of AOIs follows prior eye-tracking research in dentistry [12] and related medical domains such as chest radiography [13]. The AOI coding process is further described in our Materials and Procedures section.

### Visual search processes in radiograph interpretation

Eye tracking allows to gain insights in visual search processes of radiograph interpretation. The search model by Nodine and Kundel [14], which is derived from experts’ behavior searching for lung nodules in chest radiographs, proposes four visual search stages: First, searchers gain a global impression of the radiograph which leads to a discovery search, in which suspicious areas are scrutinized. Global impression and discovery search are characterized by short, survey fixations and high radiograph coverage. Afterward, experts apply a focal analysis with foveal verification with longer fixations on anomalies [15]. Finally, searchers perform a reflective search in which anomalies are once more visited and, if necessary, decided upon. These later two search stages are characterized by long examination fixations [14].

Outside of the domain of dentistry, there is ample support from eye tracking studies that experts develop and apply a global search when interpreting radiographs [e.g., 1, 16, 17]. According to the global search in image perception [1], experts initially scan a radiograph globally at first exposure. Through this, experts achieve an overview on potentially anomalous regions quickly, which has been originally described in mammogram diagnosis [13]. Kundel et al. [1] found the probability of correctly classifying radiographs, to be highly correlated with the time until first fixating an anomaly (time to first fixation). Another important component of visual search is focal, reflective search. In a study investigating experts diagnosing lung nodules, Drew et al. [18] found experts drilling down specific areas across multiple radiographs of the same chest to have superior diagnostic performance compared to those globally scanning radiographs individually. A systematic review of existing eye tracking research in radiology found experts’ search strategies to be best characterized by a global-to-focal search strategy as proclaimed in the search model of Nodine and Kundel [14].

However, these comparisons of different skill levels provide only indirect information about the time course of different visual search processes that occur during visual search. Specifically, the number of fixations and their average length might differ over time (e.g., fixations becoming longer and fewer in number at later trial stages as the focus shifts to individual anomalies detected during global search). Partitioning trials in equally-sized time slices and aggregating fixation measures across them allows for a temporal investigation of trials, as done in a recent study regarding the multimedia effect in multiple-choice testing [19]. The present study addresses this knowledge gap by investigating the individual stages of visual search over time.

With most studies in this field pertaining to chest radiographs and mammograms [1, 13, 14, 18], the question of how generalizable findings are to visual search in OPTs arises. As one factor, the diagnostic aims across domains can differ notably: While the visual search in chest radiographs and mammograms requires the detection of either the presence or absence of any or few anomalies (i.e. nodules indicating cancer; [2, 3]), the evaluation of OPTs requires detecting all possible anomalies out of a set of multiple and diverse anomaly categories (e.g., concerning the gums, jaw, teeth; [4]). This is especially important since undetected anomalies (false-negative errors) can lead to serious patient harm, for example, stroke caused bycarotid calcification [20].

The visual search in OPTs can thus be described as a hybrid search. Hybrid search requires individuals to hold multiple target objects (e.g., anomalies in OPTs) in working memory [21]. In the case of OPTs, diagnosticians search for a variety of different anomaly types, holding already detected anomalies in working memory while scanning the OPT for any remaining anomalies. While general rules of visual search performance may apply to radiology [22], research has not yet addressed how diagnostic performance in OPT diagnosis depends on the number and variety of anomalies in each OPT. Importantly, anomalies vary in detection difficulty, most notably through differing prevalence [23]. At the same time, multiple anomalies might exhibit specific patterns of cooccurrence or might be mentally represented in anatomical groups (e.g., dental anomalies and gum anomalies) during visual search [14]. Considering that many anomalies occur in OPTs, it is particularly interesting whether global and focal search processes also occur in hybrid search. So far, however, no study has investigated the timing of visual search with eye tracking in OPTs.

First studies in the dental domain provide evidence for global search processes of experts [5, 6], whereas another study did not find differences in markers of global search processes between more or less experienced searchers [7]. Notably, these studies investigated global processes as differences between groups of varying expertise levels, thereby neglecting the possibility that lower expertise levels could also show first stages of global search processes. The question of whether dentistry students who still develop expertise apply a global-to-focal visual search, as typically observed in experts, is yet unanswered.

This is especially important since instructing students to apply experts’ visual search strategies (e.g., global, full coverage strategies) does not necessarily improve diagnostic performance [24, 25]. While high OPT coverage thus might not suffice to improve learners’ diagnostic performance, one recent study linked the detection of low-prevalence anomalies to an increased coverage of OPTs after massed practice [26]. Still, more research is needed to understand learners’ visual search strategies during OPT diagnosis [4]. These insights into the visual search behavior of dentistry students and the question if students apply a global-to-focal visual search can be helpful for designing more informed ways of dentistry training that can then be grounded in assessing, defining, and addressing an individual student’s temporal search process.

### Cognitive load in radiograph interpretation

Next to fixation measures (e.g., fixation count and duration), one way of characterizing students’ cognitive processes and visual search strategies over the course of OPT diagnosis is through cognitive load (CL). CL can be defined as the degree to which limited cognitive resources are allocated to meet demands of a current task or behavior [27].

Eye tracking can be used to measure CL through the duration of fixations, which has been applied in human-computer interaction [28], or pupil dilation. Pupil dilation refers to the degree to which pupils contract or widen to adapt to lighting or physiological processes [29]. As pupil dilation is, among others, influenced by fatigue [30] and caffeine intake [31], it is typically measured against an individual baseline. Pupil dilation has been described as a physiological correlate of cognitively demanding operations in memory search tasks [32], language translation tasks [33], and memory encoding and retrieval [34, 35]. In medical image interpretation, pupil dilation has been linked to the difficulty of diagnostic decisions [36]. In the dental domain, pupil dilation has been reported to distinguish levels of expertise in OPT diagnosis [37].

Next to these recent findings, pupil dilation and CL are relatively new concepts to apply to radiograph diagnosis. CL can give insight into when and how learners or experts elaborate on anomalies during visual search, for example in the context of a global reflective search of the search model [14]. So far, there is a lack of research investigating the utility of pupil dilation to investigate diagnostic processes and visual search strategies (e.g., decisional processes during focal search), specifically in the domain of dental radiographs. In the case of hybrid search, where information of the visual search must be stored in working memory as in OPTs [23], CL measurements can provide further insight into why anomalies were or were not detected.

### The present study

In this study, we investigated if dentistry students’ visual search in OPTs comprises global-to-focal processes through a temporal lens. To better understand visual search strategies in dentistry students, a hitherto understudied population, we also investigated how cognitive load relates to visual search and the detection of anomalies. First, students were tasked with searching for anomalies in a given OPT. During this search phase, students’ eye movements were recorded, resulting in data on gaze measures for a period of 90 s for each OPT. The actual marking of anomalies was performed in a separate phase which was not recorded. Gaze measures during the search phase were aggregated in two time slices per OPT, which are referred to as early trial stages (first 30 s) and late trial stages (last 30 s) [11]. If dentistry students’ visual search can be characterized by a global-to-focal search strategy, early trial stages are expected to be associated with many, short fixations, indicating efforts to gain a global overview of the OPT. Conversely, late trial stages are expected to be associated with few, long fixations on the full OPT *(Hypothesis 1)*.

Building on top of recent findings linking experts’ pupil dilation with anomaly difficulty [37], pupil dilation may signal elaboration of anomalies and cognitive load during a reflective search in late trial stages as proposed by the search model of Nodine and Kundel [14]. In accordance with this model, diagnostic performance is expected to increase with more cognitive load in late trial stages during anomaly fixations. Conversely, high pupil dilation and cognitive load in early trial stages during anomaly fixations should be associated with a decrease in diagnostic performance, signaling excessive task demands or difficulty *(Hypothesis 2)*. As another measure of cognitive load, this hypothesis was also tested for mean fixation duration on anomalies.

In an exploratory, preregistered analysis, the sequential positions of OPTs were investigated as a predictor of diagnostic performance to account for possible effects of fatigue over the course of experimental sessions of around 90 minutes. Moreover, as an alternative to partitioning trials in early (first 30 s) and late (last 30 s) trial stages, aggregated fixation and CL measures were plotted across 9 equally-sized time slices for trials of 90 s to investigate students’ visual search strategies on a more fine-grained level. The research hypotheses as well as their corresponding statistical tests featured in the confirmatory analysis of this study have been preregistered (https://pada.psycharchives.org/bitstream/01200256-916c-4310-91e7-1c5dcf5f5967).

## Methods

### Participants and design

The study sample consisted of dentistry students from semesters 6 to 10 from the University of Tubingen. Students received 15€ book vouchers and individual performance feedback on their diagnostic performance as compensation for voluntary participation each semester. The data collection took place in the summer term 2017, the winter term 2017/2018, and the summer term 2018. Within the terms, students from the 6th semester were measured three times and students from the 7th semester two times (only in summer term 2017). Students were able to participate repeatedly across semesters. = The selection and sequence of OPTs remained constant across all experimental sessions in our study sample. After applying common exclusion criteria, which are described in the data analysis section, the sample comprised 107 participants and 194 measurements. Taking the first measurement of each participant, participants had a mean age of *M* = 25.25 (*SD* = 2.79) and a gender distribution of 36 males and 65 females. Demographic data of six participants were missing due to technical reasons. The present data has been partly used in other publications aimed at the evaluation of interventions to improve students’ diagnostic performance [12, 26]. In this study we used the data to analyze the data in a new way with a new purpose (see preregistration: https://pada.psycharchives.org/bitstream/01200256-916c-4310-91e7-1c5dcf5f5967).

### Compliance with ethical standards

All procedures performed in studies involving human participants were endorsed by the ethics committee of the Leibniz Knowledge Media Research Center (IWM, application LEK 2017/016) that implement recommendations of the German Psychological Association and comply with the 1964 Helsinki declaration and its later amendments or comparable ethical standards. Written informed consent was obtained from all individual participants included in the study.

### Materials and procedure

#### OPTs

The experimental stimuli were 10 OPTs that were recorded during routine checks in the university hospital. Solution templates for anomalies in these OPTs were created by two experts (a maxillofacial radiologist and a prosthodontist with both over 13 years of clinical experience). Anomalies that were declared contentious by these experts were not included in data analysis. This resulted in OPTs in the study sample having between 2 and 14 areas of interest (AOIs), corresponding to 3 to 26 anomalies (anomalous regions located very closely together were summarized into one AOI since the resolution of eye tracking does not provide the necessary precision to distinguish anomalies in such clusters). Displayed on laptops at sizes ranging between 1362–750 pixels and 1552–750 pixels, all OPTs had a sufficient clinical image quality without positioning errors.

#### Apparatus

Students’ fixation behavior was recorded through RED 250 mobile eye trackers of 250Hz from SensoMotoric Instruments (SMI^TM^). Employing the default settings of the SMI software BeGaze2, fixations were classified at a threshold of 50 ms through a velocity-based algorithm with a peak velocity of 40 /s [4, 12, 25, 26]. OPTs were displayed on laptops with a screen size of 15.6 in. and a resolution of 1920 by 1080 pixels. The testing environment was held constant at an illuminance of 30–40 lx on all laptop displays, as measured with a radiological Gossen Mavomax illuminance sensor.

#### Procedure

The data collection took place in the Tubingen Digital Teaching Lab at the Leibniz-Institut für Wissensmedien in groups of up to 30 students. After signing consent and receiving written information about the experimental procedure, students were instructed to sit comfortably and not move their head during the task; then the eye tracker was calibrated usinga 13-point calibration. After calibration, students were led through a short tutorial on how to mark anomalies with a red circle through a computer mouse controlling a digital drawing tool. Then, each trial consisted of the following procedure: Students were instructed that they would see the OPT twice, once in an *exploration phase* and once in a *marking phase*. Additionally, definitions of which anomalies to mark (requiring treatment or additional diagnostic procedures) and not mark (missing teeth, sufficient treatments, generalized horizontal bone loss, and technical artifacts) were given. Before each exploration phase, students were shown a fixation cross for 2 s which functioned as a baseline measure for pupil dilation. In the exploration phase, students were asked to search for anomalies in the OPT. Each exploration phase was 90 s long. Importantly, students received no information about the remaining time during the exploration phase and the experiment automatically proceeded after the 90 s ran out. Before the marking phase, students were reminded about which anomalies to mark and not to mark. In the marking phase, which had no time constraints, students were asked to mark the anomalies in the OPT with the drawing tool. All experimental sessions consisted of a constant sequence of 10 OPTs, that is, trials.

Demographic data (age, sex) were assessed as part of the conceptual knowledge test which was administered at the end.

### Measures

#### Diagnostic performance

For each anomaly, diagnostic performance is represented by a binary outcome measure indicating whether participants correctly circled a given anomaly (i.e., AOI) during the marking phase. These markings were saved and later rated by two independent, trained raters against the solution template created by the two experts. For anomaly detection, these two raters had a high interrater reliability (Krippendorff’s *α* = 0.97; 0.98, see [12]) based on a coding of 20% of OPTs.

#### Pupil dilation (Baselined)

As a measure of cognitive load, pupil dilation was taken from BeGaze2, which calculates the average pupil diameter in mm during a fixation. To adjust this measure to individual baselines, the recorded pupil dilation during fixations on the fixation cross at the beginning of trials was subtracted from the pupil dilation during fixations recorded during visual search. If there were multiple recorded fixations on the fixation cross prior to trials, the recorded pupil dilation was averaged across fixations to obtain a baseline. To signal that the obtained measure is representing a difference in pupil dilation, it is further explicitly referred to as Δ*APD* (difference in average pupil diameter) where appropriate. Our standardization procedure of pupil dilation followed two steps. First, *z*-scoring of pupil dilation values was performed across participants and for all fixations on screen to ease interpretation of model parameter estimates. Second, for all fixations, the (*z*-scored) pupil dilation during fixations on the fixation cross at a given trial was subtracted from all (*z*-scored) pupil dilation values during the trial. Therefore, a value of –1 in Δ*APD* can be interpreted as a pupil dilation that is one standard deviation smaller than measured at baseline.

#### Mean fixation duration

Mean fixation duration represents the average length of fixations within a given time slice in seconds. This measure was employed both as an operationalization of cognitive load during anomaly fixations as well as a representation of participants’ visual search behavior for all fixations on screen. Since logarithmized mean fixation duration appeared approximately normally distributed, this measure was log-transformed for predictive modeling.

#### Number of fixations

This measure represents how often a fixation on screen occurs within a given time slice, characterizing participants’ visual search behavior. As count data, this measure was modeled through a Poisson distribution (frequently referred to as log-linear modeling) for all fixations on screen.

## Data analysis

### Exclusion criteria

Exclusion criteria for eye movements included a deviation of more than .6 visual degrees for either the x-axis or y-axis during calibration. Additionally, sessions were excluded if the overall tracking ratio fell below 80%. One experimental session which recorded trials of longer than 90 s (most likely due to a technical error) was also excluded from data analysis.

Overall, this resulted in an exclusion of 25.29% of experimental sessions. When pairing participants by semester, there was no significant difference in diagnostic performance between included and excluded participants, except for semesters 8–10, where there was a significant advantage for included participants. After exclusion, our data comprised 329,697 unique fixations, 63,916 of which were on AOIs.

### Time slices

The exploration phase of each trial/OPT was sliced into time slices of 3 x 30 s and 9 x 10 s through self-programmed algorithms in R (R Core Team, 2021). For confirmatory analysis and predictive modeling, fixation measures in both the first 30 s of trials (early trial stages) and the last 30 s of trials (late trial stages) were aggregated. For visualizing fixation measures across more fine-grained time slices, fixation measures were also aggregated in time slices of 9 x 10 s. Finally, each trial/OPT was defined as a time slice of experimental sessions to investigate possible effects of task fatigue and OPT difficulty on diagnostic performance.

### Statistical tests and power

All data analyses were conducted with R 4.0.3 (R Core Team, 2021). Prior to data analysis, an *α* level of .05 was set for all hypothesis tests. For hypothesis testing, generalized linear mixed models were employed through the R-package lme4 v.1.1–14 [38]. Specifically, we tested differences in mean fixation duration and the number of fixations across early and late trial stages as well as an interaction of the effect of cognitive load measures across early and late trial stages on diagnostic performance. For the latter, we defined both a simple model with an interaction of pupil dilation and trial stage and a complex model with an additional interaction of mean fixation duration and trial stage. Moreover, we compared both models through likelihood-ratio testing. Random effects included the anomalies, participants nested inside cohorts, and semester count (with repeated measurements inside semesters expressed as fractions) grouped by participants. All model equations can be found in the S1 Appendix. For estimating statistical power, self-programmed simulations were run in R (1000 iterations each) and documented in the preregistration of this study [39].

## Results

### The number of fixations and average fixation duration across time (Hypothesis 1)

For Hypothesis 1, we investigated whether students’ visual search, considering fixations on anomalies and all other regions of the radiograph, could be characterized as global-to-focal, with fixations becoming less frequent and longer in late trial stages (last 30 s of trials) compared to early trial stages (first 30 s of trials). Testing this hypothesis, the main effects of trial stage on the number of fixations in a log-linear model and on logarithmized mean fixation duration in a linear model were tested. The effect of late trial stage on the logarithmized number of fixations was estimated at *β* = -0.59 (*e*^*β*^ = 0.55), *z* = -123.36, *p <* .001. Consistent with our hypothesis, the model predicts around 68 fixations to happen in early trial stages, while this quantity approximately halves in late trial stages. Trial stage also significantly contributed to explaining logarithmized mean fixation duration, *χ*^2^(1) = 127.26, *β* = 0.12 (*e*^*β*^ = 1.13), *p <* .001. In line with our hypothesis, the model predicts a mean fixation duration of 0.41 s for fixations in early trial stages, while this quantity increases by around 13% in late trial stages. An overview on all fitted model parameters can be found in S2.A and S2.B in S2 Appendix.

### The relationship between pupil dilation and mean fixation duration during anomaly fixations on diagnostic performance across time (Hypothesis 2)

Investigating the relationship between cognitive load measures and diagnostic performance across early and late trial stages, generalized linear mixed models with a binomial link function were employed. Recall that this analysis aggregates fixation features on a per-anomaly level. First, a simple model featuring baselined average pupil dilation in interaction with trial stage was estimated. Against our hypothesis, the interaction of pupil dilation and late trial stage was not significant, *β* = 0.03 (ROR = 1.03), *z* = 0.47, *p* = .639. Additionally, pupil dilation across all trial stages did not appear to be predictive of diagnostic performance, *β* = 0.04 (*OR* = 1.04), *z* = 0.97, *p* = .333. Thus, pupil dilation had no association with diagnostic performance in either the early or late trial stages. Finally, the effect of late trial stage on diagnostic performance was estimated at *β* = 0.18 (*OR* = 1.20), *z* = 2.84, *p* = .005. This means that the model estimates that anomaly fixations taking place in late trial stages are 20% more likely to be associated to an anomaly that was later correctly marked (rather than not marked).

These relationships also applied to a complex model featuring an additional interaction of mean fixation duration and trial stages. Contrary to the hypothesis, this additional interaction was also not significant, *β* = -0.05 (*ROR* = 0.95), *z* = -0.47, *p* = .637. However, mean fixation duration across all trial stages was predictive of diagnostic performance, *β* = 0.23 (*OR* = 1.26), *z* = 3.28, *p* = .001. This means that the model predicts a 26% increase in the probability of anomaly marking per second increase in mean fixation duration for corresponding anomaly fixations in both early and late trial stages. Thus, duration of fixation did not differentially affect diagnostic performance in early and late trial stages, but longer fixations were generally associated with better diagnostic performance on individual anomalies.

Comparing both models through a likelihood-ratio test, the complex model rejected the model with just an interaction of pupil dilation and trial stage, *Δχ2*(2) = 17.78, *p* < .001. However, information criteria did not indicate a clear advantage for the complex model, *AIC* = (7307.01, 7293.23), *BIC* = (7373.72, 7374.77). A model table for the complex model can be found in S2.C and S2.D in S2 Appendix.

## Preregistered exploratory data analysis

### Visual analysis of fine-grained time slices

**Fig 1:**
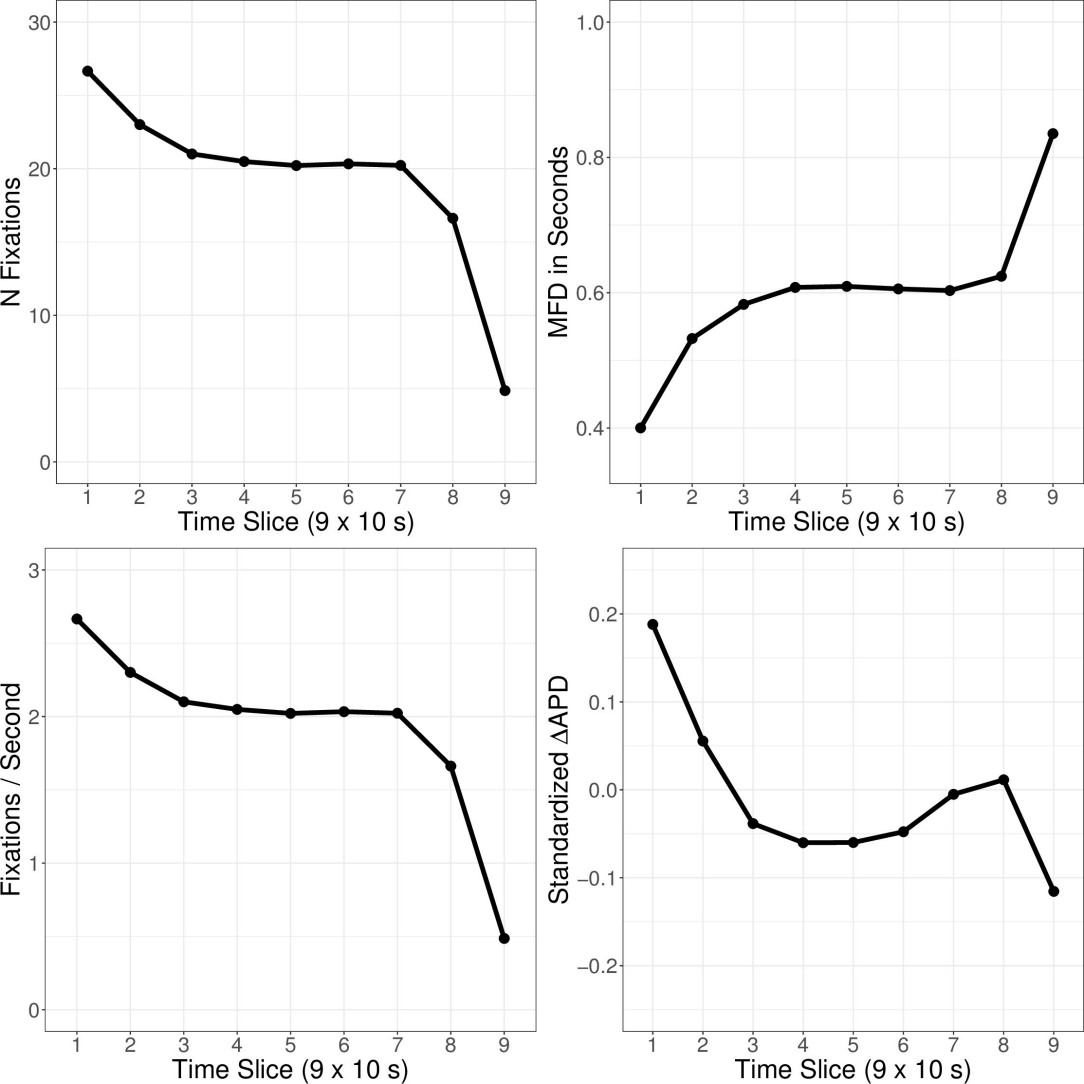
Fixation and cognitive load measures for anomaly and whitespace fixations across time (x-axis) for the number of fixations (N, top-left), mean fixation duration (MFD, top-right), fixations per second (bottom-left), and standardized, baselined average pupil diameter. (ΔAPD, bottom-right).

We investigated the general fixation behavior across all fixations in time slices of 10 s in Fig 1 to obtain a more nuanced impression of how the visual search of participants developed over time. There are two additional observations to be made: First, fixation measures seem to follow a three-stage progression, in which all measures plateau in center trial stages (time slices 4–6). Therefore, it appears appropriate to investigate the effects of these center trial stages on fixation measures and diagnostic performance in a post-hoc analysis. Second, time slice 9 (the last 10 s of trials) seems to be an outlier across all measures. While the number of fixations drastically drops in time slice 9, mean fixation duration drastically increases. At the same time, pupil dilation (as a measure of cognitive load) also decreases in time slice 9, after initially rising in time slices 7 and 8. We find that 15.89% of participants had no recorded fixation on any AOIs in time slice 9 at all.

### Sequential effects of trials and individual OPTs on diagnostic performance

**Fig 2:**
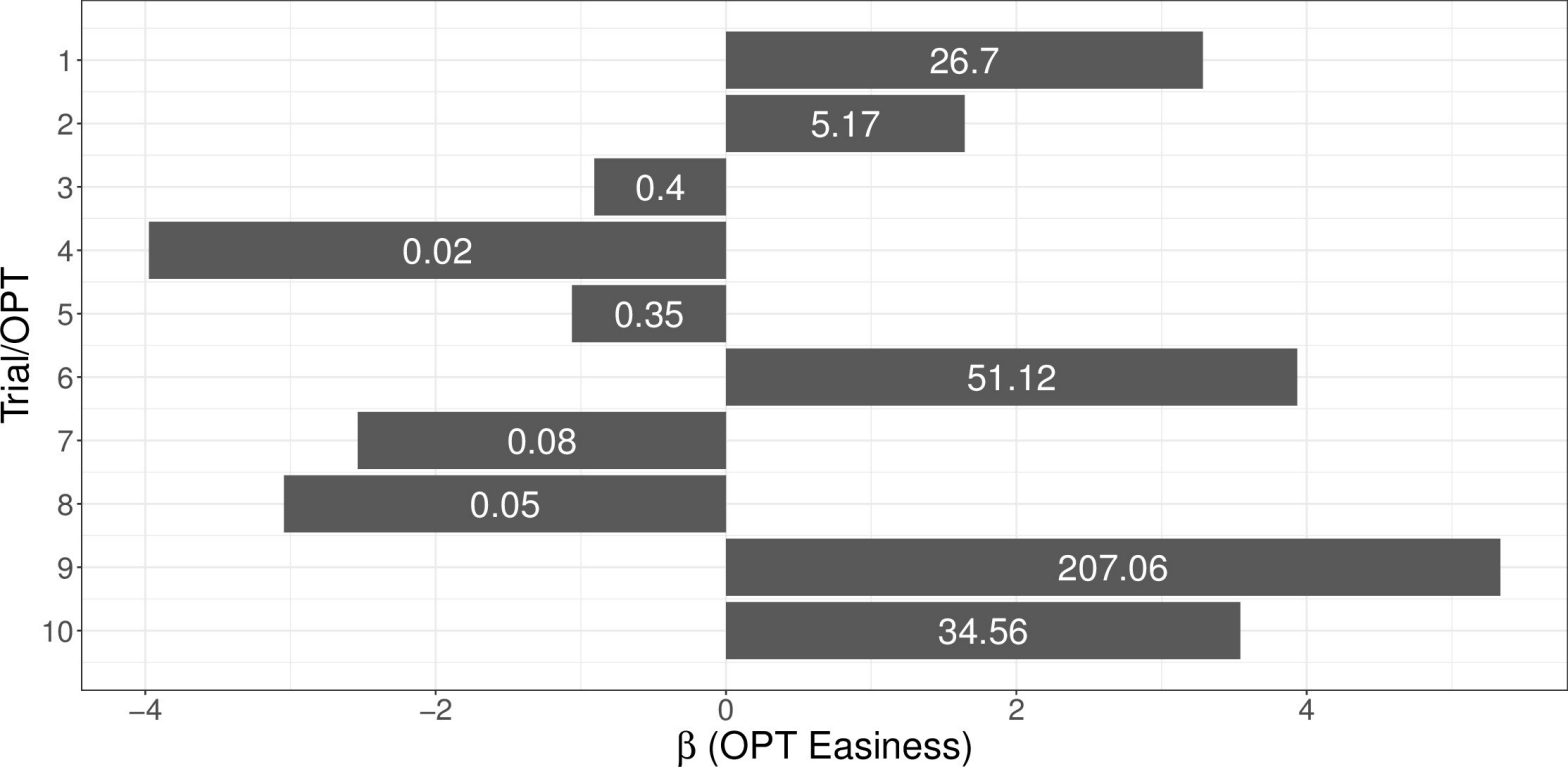
Estimated effect sizes of OPTs on diagnostic performance including odds ratios.

We investigated possible relationships between OPTs (and their sequential position) and diagnostic performance through another binomial model featuring only the OPT sequence position as fixed effects in effect coding. This allowed us to investigate effects of the sequential position and general difficulty of the OPTs (which were confounded since the sequence of OPT presentation was constant across experimental sessions). Estimated effect sizes (*ORs*) ranged from 0.03 to 61.51 (Fig 2). As an example, this means that the probability of marking an anomaly for one OPT was increased by a factor of about 62 compared to the average difficulty across all OPTs. Notably, both easy and difficult OPTs featured an average of 6.60 AOIs.

**Fig 3:**
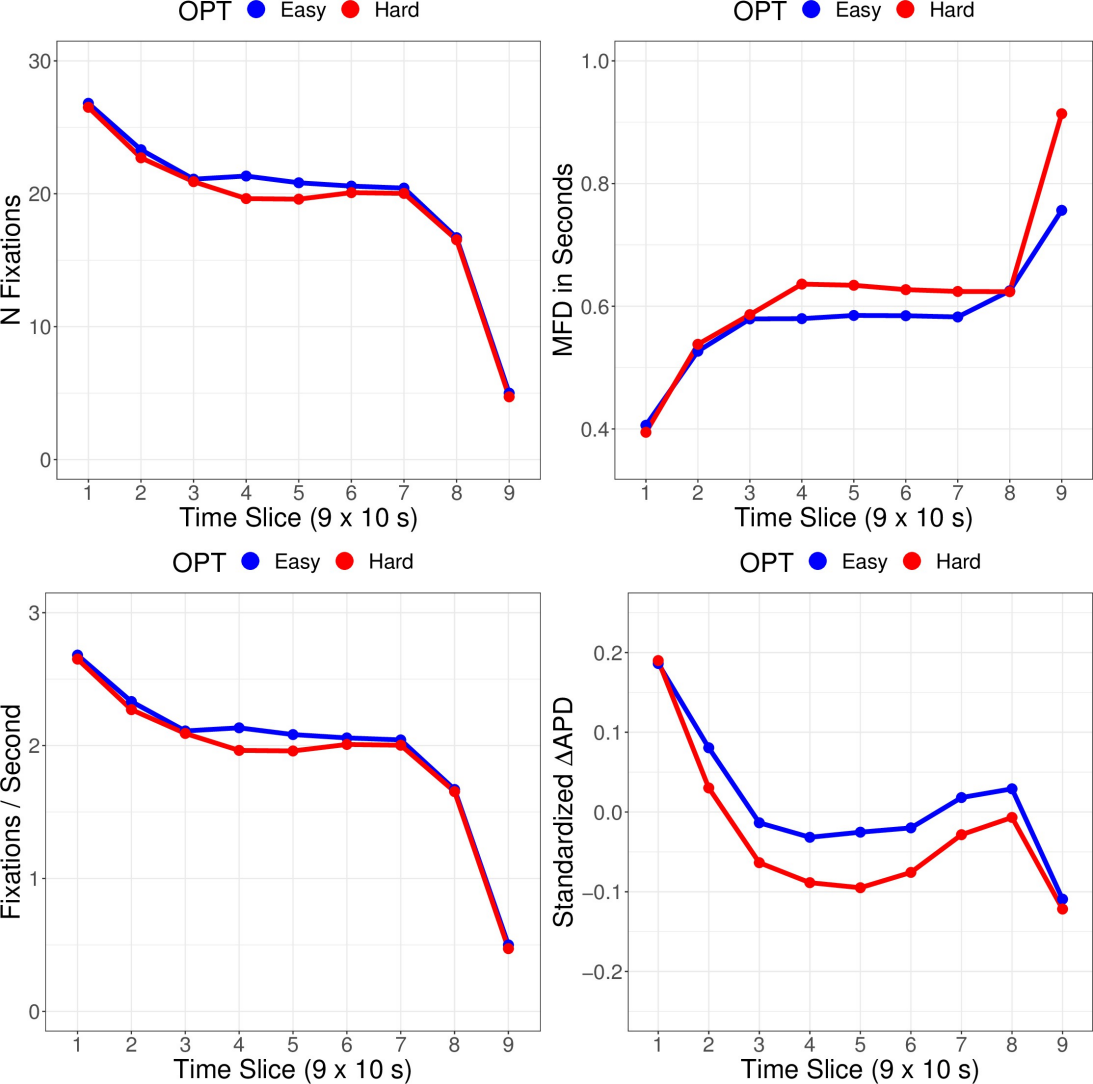
Fixation and cognitive load measures for anomaly and whitespace fixations across time (x-axis) for the number of fixations (N, top-left), mean fixation duration (MFD, top-right), fixations per second (bottom-left), and standardized, baselined average pupil diameter (ΔAPD, bottom-right) by OPT difficulty (subsets of five out of ten OPTs).

Importantly, measures of cognitive load might have low utility in particularly easy OPTs, since participants would not need to apply a lot of elaboration for successfully marking anomalies in such OPTs. This, in turn, could dilute the interaction effects of cognitive load measures and trial stage on diagnostic performance, making it harder to detect such effects in model tests. Backing up this account, there were descriptive differences in cognitive load measures between easy and difficult OPTs: We plotted general fixation measures (similar to Fig 1) across nine time slices for both the five easier and five harder OPTs in Fig 3, showing higher mean fixation duration and lower pupil dilation for difficult OPTs, particularly during center trial stages. Therefore, we decided to re-run the predictive models on diagnostic performance with just the five OPTs that were estimated to be more difficult than average, namely OPTs 3, 4, 5, 7, and 8, which had estimated *ORs* between 0.03 and 0.51 (Fig 2). The results of the post-hoc analyses are featured in the following section, which, analogous to the confirmatory analysis, revisits the two research hypotheses of this study.

### Post-hoc data analysis: The number of fixations and average fixation duration across three trials stages for difficult OPTs (revisiting Hypothesis 1)

We investigated the development of the number of fixations and mean fixation duration for all fixations on the five difficult OPTs across three trial stages of 30 s. The result was congruent with the findings from the confirmatory analysis. All fitted model parameters can be found in S2.E and S2.F in S2 Appendix.

### Post-hoc data analysis: The relationship between pupil dilation and mean fixation duration during anomaly fixations on diagnostic performance across three trial stages for difficult OPTs (revisiting Hypothesis 2)

Contrary to the results for all OPTs, pupil dilation during anomaly fixation was significantly positively associated with diagnostic performance, *β* = 0.15 (*OR* = 1.17), *z* = 2.34, *p* = .019. Additionally, the model estimated that anomaly fixations taking place in late trial stages were positively associated with correctly marking the respective anomaly, *β* = 0.23 (*OR* = 1.25), *z* = -2.55, *p* = .018.

Conducting a likelihood-ratio test, the complex model again rejected the model with just an interaction of pupil dilation and trial stage, Δ*χ*^2^(3) = 29.69, *p <* .001. This time, the gain in additional explained variance was sufficiently large enough to also lead to superior information criteria indices for the complex model, *AIC* = (4989.02, 4965.33), *BIC* = (5067.15, 5064.77). A model table for the complex model can be found in S2.G and S2.H in S2 Appendix.

## Discussion

This study aimed at investigating the visual search behavior of dentistry students during OPT diagnosis through time slice analyses. We hypothesized that the visual search behavior of students would be characterized by a global-to-focal search strategy (H1) and that cognitive load measures would be positively associated with diagnostic performance during late trial stages and negatively associated during early trial stages (H2).

In line with prior literature on experts’ global-to-focal visual search in mammogram diagnosis [1, 13] and the dental domain [6], our results support our first hypothesis on students’ visual search growing increasingly focal over time. The statistical models predicted that the number of fixations approximately halves in the last comparing to the first third of trials, while the mean fixation duration increases by around 13%. This could be explained by students initially scanning OPTs globally during the first third of trials before elaborating on anomalies during later trial stages [1, 6, 13]. This finding expands the literature through noting a global-to-focal visual search pattern for dentistry students, which systematic literature reviews described as a suitable description of experts’ visual search strategies in radiograph diagnosis [11]. Prior literature comparing levels of expertise in dentistry found mixed evidence regarding differences in global search strategies [4, 5]. We note that this might be due to a neglect of the possibility that low-expertise diagnosticians (e.g., dentistry students as featured in our sample) employ global search strategies, as found in our data. However, these results are not sufficient to evaluate the effectiveness of this strategy for students. As an example, teaching students global search strategies does not necessarily improve diagnostic performance [24].

In line with prior literature indicating that pupil dilation during visual search increases with task difficulty [40], we found that pupil dilation was only predictive of diagnostic performance in difficult OPTs. This is only partial support for our hypothesis that cognitive load measures would be predictive of diagnostic performance across trial stages (Hypothesis 2) [14]. Notably, mean fixation duration on anomalies was positively associated with diagnostic performance for all OPTs. As mean fixation duration was close to reaching a plateau in seconds 20 to 30, students might have already transitioned into a more elaborative mode of visual search during early trial stages. An alternative account would be that fixation duration and pupil dilation capture different cognitive processes. Prior work on dynamic visual memory load suggests that fixation duration is sensitive to actual memory load as well as to processing load, whereas pupil size is indicative of processing load only [41]. As we find that pupil dilation only distinguished diagnostic performance in difficult OPTs, performance differences in easier OPTs might predicated on memory load. Future work could test this hypothesis by assessing working memory as an additional covariate. Extending on [41], one could argue that pupil dilation was only predictive of diagnostic performance for difficult OPTs because anomalies in such radiographs require more elaboration to be successfully marked. On the other hand, mean fixation duration was also predictive of diagnostic performance in easier OPTs, which require less elaboration during visual search, while still allowing for attention biases that increase the probability of marking anomalies after fixating them longer than others. Controlling for false-positive errors during marking in future work could disentangle the role of attention biases during the diagnostic process. Testing the role of working memory and attentional biases in OPT diagnosis could also speak to implications for dentistry training. As our study sample is distinctly comprising students, different strategies to offload working memory (e.g., to notes) or reflect on how much time the student paid to particular anomalies via heatmaps could be potential interventions to investigate in future work.

Overall, although the analyses conducted to investigate the research hypotheses provided evidence that students were employing a global-to-focal search strategy, the results were inconsistent with some key assumptions made in the preregistration of this study [39]. Specifically, the employed division of trials in early trial stages (first 30 s) and late trial stages (last 30 s) did not capture the students’ visual search process appropriately. Rather, the visual search behavior of students followed a three-stage process. Additionally, high cognitive load during early trial stages was not associated with low diagnostic performance. Rather, cognitive load measures were already positively associated with diagnostic performance in early trial stages [37]. Notably, and contrary to our expectations, the notion that students employ a reflective search strategy during late trial stages in which they systematically revisit anomalies (as proposed by the search model of Nodine and Kundel) is not supported by the results. Most notably, this is due to students becoming increasingly selective in terms of the anomalies they fixate in late trial stages, only looking at very few anomalies during the last 10 s of trials. This might be interpreted as students allocating resources to anomalies that they are still unsure about and not decided upon. This aligns with prior literature from information problem solving, noting that fixation duration on relevant information during visual search positively correlates with problem-solving performance [42]. This finding, which differs from the model, may also be explained by the high number of anomalies in OPTs compared with few anomalies in chest radiographs, on which the model was developed. It might be easier to revisit only a few anomalies than many in the end of the visual search. On the other hand, pupil dilation drastically decreased for fixated anomalies in the last 10 s of trials. The increased mean fixation duration for the students ending their visual search and having decreased pupil dilation could, therefore, also be interpreted as an artifact of idle (thus longer) fixations. Backing up this account, we found that 15.89% of all participants never fixated anomalies in time slice 9, indicating that some students finished their visual search before the end of the trial in several instances. Still, while cognitive load measures in these last 10 s might have high utility in predicting diagnostic performance, this might only apply to a small selection of anomalies fixated in this time slice. Hence, there seems to be a trade-off between information richness (number of anomalies whose probability of marking can be informed by cognitive load measures) and levels of resolution. As an example, the described cognitive load differences in the last 10 s of trials might be diluted and difficult to detect when aggregating over time slices of 30 s, but can not be generalized to the majority anomalies since only few anomalies were fixated in the last 10 s. This trade-off is also important to consider when planning future data collections and crucial for statistical power when testing nuanced effects in fine-grained time slices of eye tracking data.

Next to these findings, the overall fit between the assumptions of the global-to-focal search model and the results of this study is remarkable given the model was originally developed in the context of visual search in chest radiographs which include diagnostic aims different from OPT visual search. While students generally shifted from a global-to-focal mode of visual search over time, one could also imagine independent, distinct search processes for individual anomalies in hybrid search. As an example, students might first search for specific types of anomalies globally, elaborate on them and then proceed with another global search for a different class of anomalies. Investigating student’s visual search for individual anomalies or classes of anomalies across time might be another promising avenue for future research.

### Limitations

This study has notable methodological limitations. First, the study sample size was ad-hoc, which limited the possibility of reliably detecting more nuanced effects of fixation measures across time. To further enhance the reliability of findings, we recommend future research to increase the sample size by recruiting more participants rather than sampling more OPTs, given the potential fatigue effects on diagnostic performance that would occur when further prolonging sessions beyond the 1 to 1.5 hours required in the present study. Future research may also include a systematic investigation of the last 10 s of trials or investigating differences in visual search strategies across time for different semesters, both being a potential inquiry for future research. Additionally, the statistical power performed in the post-hoc analysis in which difficult OPTs were systematically sampled might have been low, which has to be taken into account when interpreting the reported interaction effects of cognitive load measures and trial stage. Second, inferences made about the contribution of cognitive load measures to diagnostic performance are based on anomalies fixated by each student. At the same time, a considerable ratio of anomalies, on average, were not fixated by students. It thus remains unclear whether some classes and types (e.g., concerning the gums, jaw, teeth) of anomalies were systematically more often detected, how well the findings of this study generalize to the anomalies systematically less often detected, or whether all anomalies were detected with equal probability by students (which seems rather unlikely given the variance in OPT difficulty). Future research should critically and systematically examine different types of anomalies in OPT diagnosis. Third, the trial duration was limited to 90 seconds, and students did not know in advance when the time would end. Therefore, visual search may have been interrupted for slow students and thus would not represent the final phase of their visual search. However, we determined the time for trial duration through a pilot study and selected it so that there was sufficient time for visual search. Additionally, the data suggest that students’ visual search behavior in the last 10 seconds was very different from the time periods before that, indicating that students, on average, were not interrupted during their focal search phase.

## Conclusion and future research directions

We found that dentistry students’ visual search followed a three-stage process, becoming increasingly focal over time. At the same time, our findings do not indicate that students employ a reflective search strategy as students, which is not in line with the Nodine and Kundel model. In addition, pupil dilation was only associated with diagnostic performance for difficult OPTs. We posit that, compared to mean fixation duration, pupil dilation might capture different cognitive processes than mean fixation duration, potentially elaboration and germane cognitive load. We note that few anomalies were fixated in the last 10 s of trials, while students might have ended their visual search before the 90 s of trials ran out. Studying nuanced differences in small time slices requires a considerably higher sample size for confirmatory hypothesis testing as there appears to be a trade-off between levels of resolution and data richness. Researchers are advised to plan their sample sizes accordingly.

## Supporting information

S1 Appendix Model equations. (PDF)

S2 Appendix Model tables. (PDF)
