# Supplementary material for: A time slice analysis of dentistry students’ visual search strategies and pupil dilation during diagnosing radiographs
Source: PLoS One. 2023 Jun 8;18(6):e0283376. doi: 10.1371/journal.pone.0283376 (PMC10249848; doi:10.1371/journal.pone.0283376)
Supplement: S1 Appendix — (PDF) [file pone.0283376.s001.pdf]

S1 Appendix: Model Equations

Hypothesis 1, investigating the number of fixations (Poisson link) and logarithmized mean fixation duration (gaussian link) across time:

$$y = \beta_0 + \beta_1(trialstage) + \upsilon_i(semester|id) + \upsilon_j(cohort : id) \tag{1}$$

Hypothesis 2, investigating the relationship between cognitive load measures on diagnostic performance across time, simple model featuring baselined average pupil diameter (apd):

$$y = \beta_0 + \beta_1\beta_2(apd : trialstage) + \upsilon_i(semester|id) + \upsilon_j(cohort : id) + \upsilon_k(AOI) \tag{2}$$

Complex model also featuring mean fixation duration (mfd, trial stage abbreviated ts):

$$y = \beta_0+\beta_1\beta_2(apd : ts)+\beta_3\beta_4(mfd : ts)+\upsilon_i(semester|id)+\upsilon_j(cohort : id)+\upsilon_k(AOI) \tag{3}$$

Model equation for estimating OPT difficulty effect sizes:

$$y = \beta_0 + \beta_1(trial) + \upsilon_i(semester|id) + \upsilon_j(cohort : id) + \upsilon_k(AOI) \tag{4}$$
