# Supplementary material for: A time slice analysis of dentistry students’ visual search strategies and pupil dilation during diagnosing radiographs
Source: PLoS One. 2023 Jun 8;18(6):e0283376. doi: 10.1371/journal.pone.0283376 (PMC10249848; doi:10.1371/journal.pone.0283376)
Supplement: S2 Appendix — (PDF) [file pone.0283376.s002.pdf]

S2 Appendix: Model Tables

S2.A

Predicting (log) mean fixation duration through trial stage, confirmatory analysis:

	**Log Mean Fixation Duration**		
Predictors	*Estimates*	*95% CI*	*p*
Intercept	-0.88	-0.94 – -0.83	**<0.001**
Late Trial Stage	0.12	0.10 – 0.14	**<0.001**
**Random Effects**			
σ^2	0.11		
$\tau_{00\ cohort:id}$	0.03		
$\tau_{00\ id}$	2.77		
$\tau_{11\ id.sem}$	0.06		
$\rho_{01\ id}$	-1.00		
ICC	0.96		
N_{id}	107		
N_{cohort}	7		
Observations	3711		
Marginal R^2 / Conditional R^2	0.001 / 0.963		

S2.B

Predicting the number of fixations through trial stage, confirmatory analysis:

	**N Fixations (Poisson Link)**		
Predictors	*Incidence Rate Ratios*	*95% CI*	*p*
Intercept	68.48	64.65 – 72.53	**<0.001**
Late Trial Stage	0.55	0.55 – 0.56	**<0.001**
**Random Effects**			
σ^2	0.02		
$\tau_{00\ \text{cohort:id}}$	0.05		
$\tau_{00\ \text{id}}$	3.31		
$\tau_{11\ \text{id.sem}}$	0.07		
$\rho_{01\ \text{id}}$	-1.00		
ICC	0.99		
N_{id}	107		
N_{cohort}	7		
Observations	3711		
Marginal R^2 / Conditional R^2	0.025 / 0.995		

S2.C

Predicting diagnostic performance through trial stage in interaction with average, baselined pupil diameter during anomaly fixations (simple model), confirmatory analysis:

	**Correct Anomaly Marking (Binomial Link)**		
Predictors	*Odds Ratios*	*95% CI*	*p*
Intercept	9.40	3.32 – 26.63	**<0.001**
Avg Pupil Diameter	1.04	0.96 – 1.12	0.333
Late Trial Stage	1.20	1.06 – 1.36	**0.005**
Average Pupil Diameter x Late Trial Stage	1.03	0.91 – 1.16	0.639
**Random Effects**			
σ^2	3.29		
$\tau_{00\ cohort:id}$	0.00		
$\tau_{00\ id}$	10.77		
$\tau_{00\ bildaoi}$	17.30		
$\tau_{11\ id.sem}$	0.16		
$\rho_{01\ id}$	-0.99		
N_{id}	107		
N_{cohort}	7		
$N_{bildaoi}$	66		

Observations	12239
Marginal R^2 / Conditional R^2	0.003 / NA

S2.D

Predicting diagnostic performance through trial stage in interaction with average, baselined pupil diameter and mean fixation duration during anomaly fixations (complex model), confirmatory analysis:

	**Correct Anomaly Marking (Binomial Link)**		
Predictors	*Odds Ratios*	*95% CI*	*p*
Intercept	8.02	2.94 – 21.87	**<0.001**
Avg Pupil Diameter	1.04	0.96 – 1.13	0.296
Late Trial Stage	1.22	1.02 – 1.46	**0.026**
Mean Fixation Duration	1.26	1.10 – 1.46	**0.001**
Average Pupil Diameter x Late Trial Stage	1.02	0.91 – 1.15	0.702
Mean Fixation Duration x Late Trial Stage	0.95	0.78 – 1.16	0.637
**Random Effects**			
σ^2	3.29		
$\tau_{00\ cohort:id}$	0.00		
$\tau_{00\ id}$	11.02		

$\tau_{00\ bildaoi}$	15.95
$\tau_{11\ id.sem}$	0.16
$\rho_{01\ id}$	-0.99
ICC	0.89
N_{id}	107
N_{cohort}	7
$N_{bildaoi}$	66
Observations	12239
Marginal R^2 / Conditional R^2	0.001 / 0.891

S2.E

Predicting (log) mean fixation duration through trial stage, post-hoc analysis for three trial stages and based on difficult OPTs only:

	**Log Mean Fixation Duration**		
Predictors	*Estimates*	*95% CI*	*p*
Intercept	-0.89	-0.95 – -0.84	**<0.001**
Center Trial Stage	0.17	0.14 – 0.20	**<0.001**
Late Trial Stage	0.13	0.10 – 0.16	**<0.001**
**Random Effects**			
σ^2	0.12		

$\tau_{00\ cohort:id}$	0.03
$\tau_{00\ id}$	1.98
$\tau_{11\ id.sem}$	0.04
$\rho_{01\ id}$	-1.00
ICC	0.94
N_{id}	107
N_{cohort}	7
Observations	2798
Marginal R^2 / Conditional R^2	0.002 / 0.943

S2.F

Predicting the number of fixations through trial stage, post-hoc analysis for three trial stages and based on difficult OPTs only:

	**N Fixations (Poisson Link)**		
Predictors	*Incidence Rate Ratios*	*95% CI*	*p*
Intercept	68.73	65.13 – 72.52	**<0.001**
Center Trial Stage	0.85	0.84 – 0.86	**<0.001**
Late Trial Stage	0.55	0.54 – 0.56	**<0.001**
**Random Effects**			
σ^2	0.02		

$\tau_{00\ cohort:id}$	0.04
$\tau_{00\ id}$	2.93
$\tau_{11\ id.sem}$	0.06
$\rho_{01\ id}$	-1.00
$N_{\ id}$	107
$N_{\ cohort}$	7
Observations	2798
Marginal R^2 / Conditional R^2	0.777 / NA

S2.G

Predicting diagnostic performance through trial stage in interaction with average, baselined pupil diameter during anomaly fixations (simple model), post-hoc analysis for three trial stages and based on difficult OPTs only:

	**Correct Anomaly Marking (Binomial Link)**		
Predictors	*Odds Ratios*	*95% CI*	*p*
Intercept	9.32	1.90 – 45.78	**0.006**
Avg Pupil Diameter	1.17	1.03 – 1.33	**0.019**
Center Trial Stage	1.18	1.00 – 1.40	0.053
Late Trial Stage	1.25	1.04 – 1.51	**0.018**
Average Pupil Diameter x	0.88	0.73 – 1.06	0.168

Center Trial Stage			
Average Pupil Diameter x Late Trial Stage	1.06	0.86 – 1.30	0.608
**Random Effects**			
σ^2	3.29		
$\tau_{00\ cohort:id}$	0.00		
$\tau_{00\ id}$	16.45		
$\tau_{00\ bildaoi}$	20.42		
$\tau_{11\ id.sem}$	0.30		
$\rho_{01\ id}$	-0.99		
N_{id}	107		
N_{cohort}	7		
$N_{bildaoi}$	33		
Observations	8979		
Marginal R^2 / Conditional R^2	0.008 / NA		

S2.H

Predicting diagnostic performance through trial stage in interaction with average, baselined pupil diameter and mean fixation duration during anomaly fixations (complex model), post-hoc analysis for three trial stages and based on difficult OPTs only:

	**Correct Anomaly Marking (Binomial Link)**		
Predictors	*Odds Ratios*	*95% CI*	*p*
Intercept	8.14	1.65 – 40.29	**0.010**
Avg Pupil Diameter	1.17	1.03 – 1.33	**0.018**
Center Trial Stage	1.02	0.80 – 1.31	0.844
Late Trial Stage	1.28	0.99 – 1.66	0.056
Mean Fixation Duration	1.26	1.04 – 1.53	**0.020**
Average Pupil Diameter x Center Trial Stage	0.88	0.74 – 1.06	0.191
Average Pupil Diameter x Late Trial Stage	1.05	0.85 – 1.29	0.638
Mean Fixation Duration x Center Trial Stage	1.20	0.91 – 1.57	0.191
Mean Fixation Duration x Late Trial Stage	0.95	0.73 – 1.25	0.723
**Random Effects**			
σ^2	3.29		

$\tau_{00\ cohort:id}$	0.00
$\tau_{00\ id}$	16.26
$\tau_{00\ bildaoi}$	20.51
$\tau_{11\ id.sem}$	0.29
$\rho_{01\ id}$	-0.99
N_{id}	107
N_{cohort}	7
$N_{bildaoi}$	33
Observations	8979
Marginal R^2 / Conditional R^2	0.020 / NA
